# Contemporary approach to desensitization: Targeted therapies for HLA sensitized pediatric heart transplant candidates – Study design and rationale

**DOI:** 10.1016/j.jhlto.2025.100429

**Published:** 2025-12-05

**Authors:** Lakshmi R. Gokanapudy Hahn, Marlena Habal, Adriana Zeevi, Kathleen E. Simpson, Warren Zuckermann, Kevin Daly, Chad Mao, Joseph Rossano, James K. Kirkin, Charles E. Canter

**Affiliations:** a Washington University in St. Louis, School of Medicine, St. Louis, MO; b NYU Grossman School of Medicine, New York, NY; c University of Pittsburgh, Pittsburgh, PA; d University of Colorado Anschutz Medical Center, Aurora, CO; e New York Presbyterian Hospital – Columbia University Medical Center, New York, NY; f Boston Children’s Hospital, Boston, MA; g Children’s Healthcare of Atlanta, Atlanta, GA; h The Children’s Hospital of Philadelphia, Philadelphia, PA; i Kirklin Solutions, Birmingham, AL

**Keywords:** Desensitization, Pediatrics, Heart transplant, Carfilzomib, Belatacept

## Abstract

**Purpose:**

HLA sensitization significantly limits donor availability and increases waitlist mortality in pediatric heart transplantation (HT). Current desensitization strategies are largely ineffective or equivocal. Recent adult studies show that a dual approach with Carfilzomib (CFZ), a proteasome inhibitor, and Belatacept (BELA), a costimulation blocker, reduces class I and II HLA antibodies (Abs). This study will evaluate the clinical utility of CFZ and BELA in reducing HLA antibody type and strength in pediatric and young adult patients.

**Methods:**

This prospective, observational study will include about 30 patients from 6 pediatric clinical sites, an HLA core and a mechanistic core lab. Patients aged 10–24 years, highly sensitized with class I and/or class II cPRA ≥ 50% (MFI > 4000), will be included. Exclusion criteria: EBV seronegative, HIV+, and a history of hematologic malignancy. Secondary endpoints include mechanistic studies on how desensitization affects cellular subsets producing HLA Abs, whether reductions in antibody strength persist until transplant, and post-transplant outcomes such as antibody mediated rejection and graft survival.

**Results:**

The study’s unique design harmonizes the use of a novel protocol across sites using a central IRB and is the first prospective, registry-based investigation in pediatric HT using the Pediatric Heart Transplant Society (PHTS) registry. The HLA core will measure antibody response through MFIs, titers, and cPRA, while the mechanistic core will use advanced investigations to support the trial's clinical endpoints.

**Conclusion:**

This multicenter study aims to establish a transformative, standardized approach to desensitization and antibody evaluation.

## Introduction

The presence of antibodies (Ab) targeting non-self human leukocyte antigens (HLA), also known as HLA sensitization, is a major barrier to heart transplantation (HT) in pediatric and young adult patients, increasing both wait times and waitlist mortality^1^. Attesting to the gravity of this problem, an analysis of all children listed for isolated HT from 1995 to 2009 in the USA found that requiring a negative prospective crossmatch (-XM) increased waiting time and more importantly increased waitlist mortality^2^. The Clinical Trials in Organ Transplantation in Children (CTOTC-04) study observed that PHT across a +XM, in sensitized patients (MFI > 1000) was associated with acceptable first year graft and patient survival, although AMR rates were high and correlated with higher DSA strength^3^. A recent analysis of the PHTS Registry found that of the 4599 pediatric heart transplants (PHT) performed between 2010–2021, 9.5% of transplants were performed across a +XM. No significant difference was noted in 10-year survival between the +XM vs. -XM groups, however, a cPRA > 50% in the +XM group emerged as a significant risk factor for graft loss^4^. A recent analysis of the United Network for Organ Sharing (UNOS) database found that both 1-year mortality (nearly 30%, a 4-fold increase) and graft loss were higher in highly sensitized (cPRA > 80%) pediatric patients compared to non-sensitized patients, and +XM was also associated with increased risk of mortality and graft loss^5^. Collectively, sensitization reduces access to transplant by limiting the donor pool and +XM in patients who are highly sensitized (cPRA > 50%) shortens post-transplant graft survival defining an unmet need.

**Figure 1:**
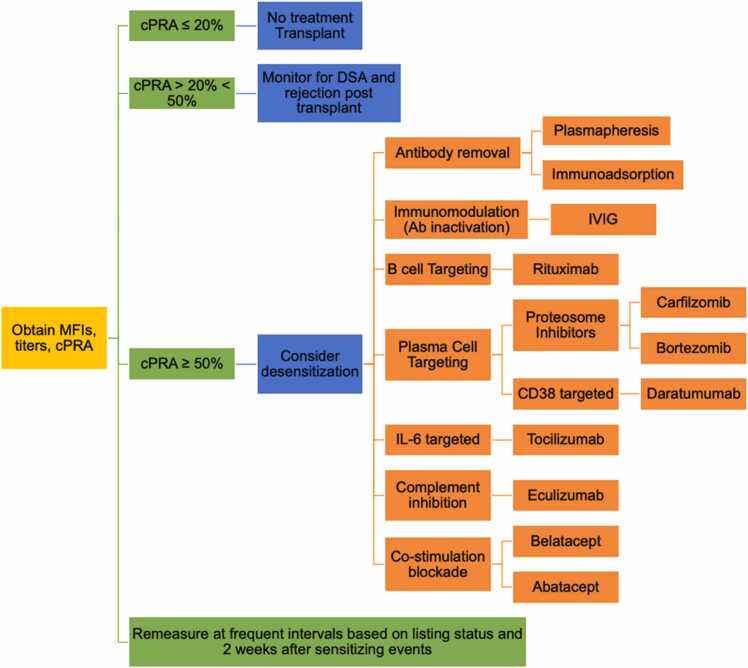
Conceptual schema for a sensitized patient. Desensitization therapy is considered for patients with strongly binding anti-HLA antibodies and a calculated panel-reactive antibody (cPRA) of greater than 50%.

Despite these consequences, there is no standard-of-care approach to the sensitized PHT candidate. Importantly, desensitization is used less commonly than in the adult population, with only 25% of the patients in the above cohort receiving desensitization treatment. The implications of anti-HLA Abs on transplantation vary based on antibody strength as defined by mean fluorescence intensity (MFI) and antibody titer^6^. The presence of HLA Ab is determined by the Luminex single antigen bead (SAB) assay; we will define MFI in titer as a surrogate for antibody strength. Access to the donor pool is determined by entering unacceptable antigens based on center specific MFI cut off values into the online UNOS (United Network for Organ Sharing) calculator to determine the calculated panel-reactive antibodies (cPRA). The percent cPRA informs the clinical team as to the degree of sensitization, with a higher cPRA further limiting the donor pool (Figure 1). For example, a cPRA_MFI>4000_≥ 50% indicates that >50% of donors would be unacceptable because they have HLA antigens to which the HT candidate has HLA antibodies at levels > 4000 MFI**.** The change in cPRA on neat (undiluted) serum is an insensitive measure of effectiveness, and instead, the circulating HLA Ab should be risk stratified by performing serial dilutions with cPRA reduction calculated per antibody titer^7^, ^8^. This approach allows the clinical team to differentiate between HLA antigens that should be avoided from those that are potentially acceptable to cross. Dilutional analysis can act as a surrogate for complement-binding capacity (e.g., C1q positivity), and reduction in cPRA at lower titer thresholds has been associated with improved transplant outcomes.

For highly sensitized candidates, various desensitization strategies can be employed in an effort to eliminate or decrease the strength of anti-HLA Ab, thereby decreasing the cPRA, increasing the donor pool, and maximizing the likelihood of transplantation. Efficacy of desensitization can be assessed based on percent of antibodies that drop to low titer (<1:16) post-treatment^9^. Desensitization strategies, however, are used rarely and have unclear efficacy^10^, ^11^, ^12^, ^13^, ^14^. Distinct from adult transplant candidates, pediatric candidates are i) more likely to have congenital heart disease, where exposure to bypass and homograft during prior palliative procedures markedly increases the risk of sensitization, and ii) less likely to benefit from long-term durable support, together underscoring the urgent need to define pathways to transplant for these patients. No standardized management protocols for desensitization in pediatric HT (PHT) exist to date.

## Desensitization strategies

Multiple strategies have been used to treat sensitization in HT candidates, mainly adapted from the renal transplant experience (Table 1). These therapies focus on multiple targets of the humoral immune pathway to either achieve a -XM pretransplant or to reduce the impact of DSA in +XM transplant. At low titer, antibody reduction can be achieved by removal with plasmapheresis and or inactivation with use of intravenous immunoglobulin (IVIG). The use of B cell depleting agents (rituximab), inhibitors of complement activation (eculizumab), inhibitors of IL-6 (tocilizumab) and monoclonal antibody against CD38 (daratumumab) remain more experimental and limited to highly sensitized patients. The current literature in adults is not abundant, mostly observational, with small cohorts, short follow-up, and with inconsistent treatment methodologies. Current pediatric literature is even more limited and efficacy of desensitization therapies in pediatric HT recipients as of yet remains unclear. While there are some studies reporting success in facilitating HT, many pediatric patients do not respond, or experience Ab rebound, highlighting the diversity of the individual patients’ immune response^10^, ^11^, ^12^, ^13^, ^14^, ^15^. Hence, there is an urgent need to standardize management protocols across centers so as to i) advance our knowledge thereby ii) informing treatment decisions for this high-risk group of patients.

**Table 1:** Current Strategies for Desensitization of Pediatric Heart Transplant Candidates

Drug/MOA	Summary	Limitations/Concerns
**IVIG**		
Derived from the gammaglobulin fraction of pooled human plasma. Has multiple immunomodulatory effects: neutralizes circulating antibody, inhibits complement and inhibits B cell activation and maturation through upregulation of inhibitory B-cell FcγRIIB.	Most commonly used desensitization therapy in pediatric heart transplantation, included in upto 79% of protocols. Small studies show it can reduce PRA and enable transplantation, particularly when used in combination with plasmapheresis.	Effectiveness is variable – especially when assessed with sensitive assays like single antigen bead, and cPRA reduction is often modest. IVIG is generally more effective as part of a multimodal approach rather than monotherapy.
**Plasmapheresis**		
Removes circulating immunoglobulins via extracorporeal plasma antibody filtration.	Often used alongside IVIG, has been shown to reduce PRA in some heart transplant candidates through outcomes vary by antibody burden and patient factors.	Antibody rebound due to rapid diffusion from extravascular space can occur and multiple treatments are usually needed to achieve low circulating antibody levels.
**ANTI CD20**		
**Rituximab**		
A chimeric murine/human monoclonal IgG1 antibody directed against CD20, expressed on mature B cells. Near complete depletion of peripheral blood B cells is achieved through a combination of antibody dependent cellular cytotoxicity (ADCC), complement-dependent cytotoxicity, and apoptosis.	Studies in both pediatric^16^ and adult heart transplant patients have noted cautiously optimistic results, especially when Rituximab was combined with IVIG. However, the non-randomized clinical experience has usually been heterogenous and particularly in the renal RCTs has been inadequate.	Limited ability to adequately suppress HLA Ab responses since: 1. It does not prevent denovo DSA. 2. CD20 Ag is absent on B cell precursors and antibody secreting plasmablasts/ plasma cells. 3. It incompletely eliminates CD27+ memory B cells
**PLASMA CELL INHIBITORS**		
**Bortezomib**	(described in detail in manuscript)	
**Carfilzomib**		
**Daratumumab**		
A high affinity human IgGκ monoclonal antibody that targets CD38 and induces PC apoptosis via Fcγ receptor mediated cross-linking and macrophage mediated phagocytosis. It also promotes expansion of memory and naïve T-cells^17^.	Its success in treating myeloma and AL amyloidosis has led to its off-label use in transplant settings for desensitization and AMR. CD38 is also expressed on natural killer (NK) cells, which may provide additional benefit, particularly in the setting of AMR and chronic rejection. Early case series and small trials predominantly in kidney transplantation suggest it can significantly reduce DSAs and stabilize or reverse AMR^13^. In pediatric patients, experience with daratumumab is even more limited, with only isolated case reports^18^^,36,37^ and small series that suggest potential efficacy with a decline in cPRA.	1. The efficacy may be limited by the persistence of long-lived plasma cells within bone marrow niches that can continue producing alloantibodies despite treatment. 2. Immune reconstitution could lead to the rebound effect described in the case series. 3. Since CD38 is also expressed on multiple suppressor cell lineages, there is a possibility of T cell mediated rejection following Daratumumab.
**COSTIMULATORY BLOCKADE**		
**Belatacept**	(described in detail in manuscript)	
**ANTI-IL6/IL6R**		
**Tocilizumab**		
Anti-IL6 receptor.		
Broad effects on B cell maturation, T cell activation and germinal center responses.	Have shown modest antibody reductions in highly sensitized kidney transplant candidates, especially when combined with IVIG. Outcomes vary, particularly in patients with high-titer DSA.	Limited effect as monotherapy since they are not robust inhibitors or depleting agents, thus unable to significantly alter the immune response.
**Clazakizumab**		
Direct IL6 blocker		
**COMPLEMENT INHIBITORS**		
**Eculizumab**		
Humanized anti-CD5 monoclonal antibody. Inhibition of C5 cleavage to C5a and C5b prevents the formation of C5b-9 macrophage activating complex (MAC) and its downstream effects.	Approved for the treatment of paroxysmal nocturnal hemoglobinuria and atypical hemolytic uremic syndrome, and has been used in transplantation to treat refractory antibody mediated rejection. Patel et al led a prospective trial of terminal complement inhibition after high immunologic risk heart transplantation and found that it was well tolerated with favorable post transplant outcomes. When compared with patients treated with plasmapheresis and IVIG, eculizumab was associated with dramatic decrease in incidence of biopsy proven AMR.	

## Carfilzomib and belatacept

Desensitization therapies as discussed above, generally deplete or modulate antibodies or affect B-cell activity without affecting antibody production by plasma cells. Proteosome inhibitors (PIs) induce apoptosis in response to the accumulation of misfolded proteins. The high rate of immunoglobulin synthesis by antibody secreting cells (ASCs) underlies their susceptibility to PI-based therapies. Carfilzomib (CFZ) is a 2nd generation, irreversible inhibitor of 20S proteasomal subunit. CFZ causes less neuropathy than its first-generation cousin bortezomib and may have less antibody rebound due to its irreversible binding^11^. CFZ as desensitization monotherapy in renal transplant patients led to a reduction in HLA antibodies and in bone marrow plasma cells (BMPCs) with acceptable drug safety and toxicity^16^. In HT candidates, desensitization with CFZ, plasmapheresis and IVIG led to a decrease in cPRA from 76% to 40%. A rejection episode was documented in a patient with C1q-PRA of 54% suggesting a limitation in more highly sensitized candidates^15^. From a mechanistic standpoint, bone marrow CD138+ plasma cells (PCs) secrete antibodies with specificities that mirror those in peripheral blood – underscoring their contribution to HLA sensitization. CFZ reduces CD138+ BMPCs by ≥ 50%, including the HLA repertoire in sensitized patients^17^. However, the *response is incomplete* and accompanied by brisk rebound following treatment completion^16^. While several factors are thought to contribute, homeostatic proliferation in germinal centers (GCs) is thought to drive repopulation^18^, highlighting the contribution of memory B cells, which can enter the GC leading to robust recall responses. These observations argue for combinatorial therapies targeting not only the PC compartment but also GCs and memory B cells.

The T-dependent nature of the anti-HLA antibody response suggests T-cell/B-cell interactions as a rational therapeutic target. The critical interactions include CD28:CD80/86, CD40:CD40L, and ICOS:ICOSL; and the ability to block these is collectively termed costimulation blockade^19^. The major clinical target to date has been the CD28:CD80/86 pathway and this has been achieved using the fusion protein CTLA4-Ig (abatacept) and its high affinity variant belatacept^20^. Belatacept (BELA) is a humanized IgG1 antibody against CD80/CD86 fused with CTLA4 that outcompetes T cell CD28 for interactions with CD80/86 found on antigen-presenting cells. BELA has been approved for use in renal transplant recipients on the basis of two randomized controlled trials, where compared with calcineurin inhibitor (CNI) based immunosuppression, it was associated with strikingly low incidence of *de novo* DSA and superiority in constraining pre-existing HLA Ab responses^21^, ^22^. Evidence suggests that this may be attributed to the ability of BELA to blunt germinal center (GC) responses^23^, ^24^, ^25^, ^26^. BELA has been used in adolescents and children as young as 3 years old following renal transplantation and found to be well tolerated other than some episodes of clinical neutropenia^27^.

### Study design

This is a prospective, multicenter, observational study of a dual immunotherapy approach for desensitization that will combine an intensive PI, CFZ based regimen with costimulatory blockade with BELA. This strategy is novel in PHT patients and we hypothesize that it will substantially enhance our ability to transplant highly sensitized (cPRA_MFI>4000_ ≥ 50%) pediatric/ young adult patients.

#### Primary aim

Study the efficacy and effectiveness of our proposed combined protocol with CFZ and BELA. We will investigate whether each cycle of desensitization is effective at.

i) reducing antibody strength as measured by MFI and titers and.

ii) lowering the calculated panel reactive antibody (cPRA).

We hypothesize that this protocol will lead to a meaningful decrease in antibody strength, with stepwise dilution of serum demonstrating gradual elimination or decrease in MFI such that the corresponding antigens would no longer be considered unacceptable. This would lower the cPRA, thereby allowing the use of a greater donor population. We will also determine whether the reduction in antibody strength is maintained with BELA between the last cycle and time of transplant. Reduction in DSA at the time of crossmatch will be studied to establish the functional relevance of our strategy. Sensitizing events defined per CTOT criteria^3^, as well as clinical events associated with the use of the proposed protocol will be monitored.

#### Secondary aim 1

Determine how desensitization impacts cellular subsets responsible for circulating anti-HLA antibodies. Using a series of state-of-the art techniques, we will combine global approaches (multiparameter flow, transcriptomics) with studies to delineate antigen specific responses (allo and viral) together establishing how desensitization shapes the B- and T-cell compartment and determine whether the response is sustained under BELA.

#### Secondary aim 2

Assess waitlist mortality and post-transplant outcomes using the Pediatric Heart Transplant Society (PHTS) database. We hypothesize that mortality before and after transplant will be comparable to overall PHTS outcomes^5^, both with and without positive crossmatch. Crossmatch (XM) status at the time of transplant will be recorded for each patient, and patients will be followed for at least one year after transplant to assess for antibody mediated rejection (AMR), ISHLT grade acute cellular rejection ≥2R (ACR), cardiovascular mortality, or need for retransplantation. While the primary analysis will focus on the first post-transplant year to align with available PHTS data, we anticipate extending this work in the future to evaluate longer term outcomes as data maturity allows.

Additional PHTS defined outcomes including major infections within the first post-transplant year will also be analyzed, given their relevance to overall morbidity and mortality in this population. Although development of de novo donor specific antibodies (DSA) is not explicitly captured within PHTS, this represents a key mechanistic endpoint linking sensitization to AMR and mortality. If feasible, we will explore integration of site level DSA data or use donor derived cell free DNA (dd cfDNA), which is available through PHTS, as a surrogate marker of alloimmune injury.

### Study rationale

Early work in mouse models demonstrated that CTLA4-Ig could inhibit the memory B cell response, collapse ongoing GC B cell reactions and stop the rise in alloantibody following HT^28^. The addition of bortezomib to a continuous CTLA4-Ig regimen, but not bortezomib alone, led to sustained alloantibody suppression which mirrored the clinical response in a small cohort of renal transplant recipients^25^. The studies described thus far highlight that plasma cell directed therapies as part of desensitization regimens are limited by i) inadequate response, particularly in very highly sensitized candidates, ii) antibody rebound/PI resistance and iii) their side effect profile^13^. Rigorous non-human primate studies have shown that desensitization using proteosome inhibition (PI) and costimulatory blockade with Belatacept (BELA) as a ‘dual targeting approach’ reduces BMPCs, disorganizes GC responses, reduces DSA levels and prolongs allograft survival^29^, ^30^.

### Preliminary studies

Habal et al translated these findings into the clinical setting for 4 highly sensitized (cPRA > 99%, C1q+) adult HT patients in whom the combination of BELA/PI significantly reduced both class I and class II HLA Abs, increased the likelihood of identifying an acceptable donor and led to sustained post-transplant suppression of DSA^12^. Preliminary findings suggest that the reduction in HLA Abs may be sustained with ongoing monthly BELA infusion. This is supported by observations in renal transplant patients as discussed above^21^.

At Washington University in St. Louis (WashU), St. Louis Children’s Hospital (SLCH), we have extended this to the pediatric population performing what is to our knowledge the first case of desensitization with BELA and CFZ in a highly sensitized pediatric patient (17 years old). The patient experienced a brisk response to one cycle of treatment bringing cPRA_MFI>4000_ down from 47% to 16%. The effectiveness and efficacy of this dual targeting approach with CFZ and BELA therefore warrants further study to determine clinical utility in pediatric and young adult patients.

### Collaboration and data coordination

This study represents a unique collaboration between six high-volume PHTS sites with prior experience in multicenter consortia such as CTOT. Through a formal partnership with PHTS, the registry infrastructure will be leveraged for the first time to support a prospective, multicenter investigation. All enrolled patients, including those older than 18 years, will be entered into PHTS by their corresponding sites and assigned a unique study identifier.

Each site will collect and process clinical and laboratory data according to the standardized study protocol. Samples will be logged, processed, stored, and shipped to two central cores: the mechanistic core (New York University, PI MH) and the HLA core laboratory (University of Pittsburgh, PI AZ). Mechanistic and immunologic assay results will be submitted to WashU using standardized reporting templates.

PHTS will transmit compiled clinical event data from participating sites to Washington University using the same unique identifiers, enabling secure linkage of clinical, mechanistic, and HLA data. Kirklin Solutions and WashU will jointly serve as the Data Coordinating Center (DCC), with primary responsibility for data management, site monitoring, adjudication, statistical analysis, and preparation of abstracts and reports. All final statistical analyses will be conducted at WashU.

### Single institutional review board approval

WashU serves as the single institutional review board (sIRB) for the study. All participating sites have chosen to execute a reliance agreement with the WashU IRB for primary review of the protocol. Site-specific consent form modifications were incorporated as needed, such as language surrounding institutional requirements for HIPAA, and age of assent which may differ between sites. This clinical study is being conducted using good clinical practice (GCP), as delineated in Guidance for Industry: E6 Good Clinical Practice Consolidated Guidance 1, and according to the criteria specified in this study protocol.

### Known and potential risks

Risks associated with BELA: In the BENEFIT trial, a higher incidence of posttransplant lymphoproliferative disease (PTLD) was observed in BELA treated renal transplant recipients who were naïve for prior Epstein Barr virus (EBV) infection at time of transplant^31^. As such, we will exclude patients who are EBV seronegative. There is emerging evidence of BELA associated risks of greater CMV morbidity^32^.

Risks associated with CFZ: New onset or worsening of pre-existing cardiac failure, acute renal failure and pulmonary toxicity have been reported. Adverse reactions could include anemia, diarrhea, hypertension, fatigue, thrombocytopenia, peripheral neuropathy and dyspnea.

### Study population

Inclusion criteria: Patients between 10 and 24 years old, who are being considered for HT and found to be highly sensitized with class I and or class II cPRA ≥ 50% utilizing the MFI threshold of 4000 (cPRA_MFI>4000_ ≥ 50%) will be considered for this desensitization strategy.

Exclusion criteria: EBV seronegative; HIV+; history of hematologic malignancy; inability to give a written informed consent or comply with study protocol.

### Number of subjects and sample size justification

A cohort of 30 patients across the 6 clinical sites during the 4-year study period is fixed by the limited population of patients meeting eligibility criteria for the study. This study is preliminary and results from the current work will be used to inform reasonable effect sizes when calculating power and sample sizes for a larger-scale study. With a cohort of 30 patients, we will have 80% power to detect moderate standardized effect sizes of Cohen’s d = 0.53 for analyses using paired t-tests and Cohen’s d=0.55 for analyses using Wilcoxon Signed Rank tests (Aim 1). We will also have 80% power to detect a large Chi-square effect size of W=0.51 (Aim 3).

### Rationale for why an IND is not needed

CFZ and BELA are two lawfully marketed drugs utilized for desensitization in medical practice, especially in patients who are highly sensitized. The intent in these scenarios is to treat the patient and attempt to increase the donor pool so that the patient can undergo heart transplantation. We aim to observe how these drugs impact the HLA antibodies, as determined by a core laboratory. This is not a clinical trial, and we do not intend to study a new indication, change the labeling or advertising of the drug. The study does not involve a route of administration, dose, patient population or other factor that significantly increases the risk (or decreases the acceptability of the risk) associated with the use of the drug products. The study will be conducted in compliance with the requirements for review by the IRB and with requirements for informed consent. The study is not intended to promote or commercialize the drug products. This approach complies with the FDA's "Guidance for Clinical Investigators, Sponsors and IRBs: Investigational New Drug Applications (INDs) – Determining Whether Human Research Studies Can be Conducted Without an IND.".

### Study regimen

**Figure 2:**
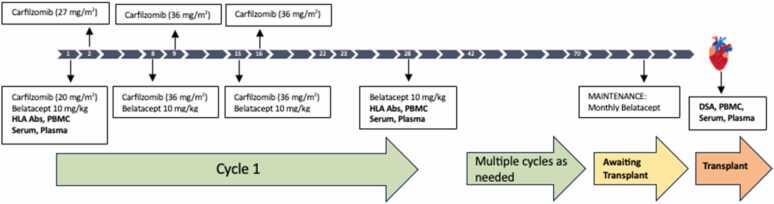
Desensitization Protocol using a combination of carfilzomib and belatacept. Human Leukocyte Antigen antibodies (HLA Abs); Peripheral Blood Mononuclear cells (PBMCs); Donor Specific Antibodies (DSA).

The desensitization protocol is a combination of CFZ and BELA to be given every week for 4 weeks as part of one cycle (Figure 2). CFZ 20 mg/m^2^ IV on day 1, 27mg/m^2^ on day 2, and 36 mg/m^2^ on days 8, 9, 15, and 16. Patients will receive pre-medication with acetaminophen, diphenhydramine, methylprednisolone and ondansetron to minimize side effects. Patients will also be pre-hydrated with 500 ml normal saline prior to CFZ based on clinical status. BELA 10 mg/kg on days 1, 8, 15, and 28, and q28 days after first cycle; to be infused over 30 min using a 0.2-to-1.2-micron low protein binding filter. We anticipate that a number of patients will need multiple cycles in order to have sequential reduction in antibodies and to increase the likelihood of finding a donor. Once patients are deemed to be adequately desensitized (see Antibody evaluation), they will be maintained on monthly BELA (10 mg/kg) until the time of transplant. Following cycle 1, centers can choose to augment therapy (for example, with plasmapheresis) based on their established desensitization protocols and clinical judgment.

### Antibody evaluation

We will collect samples pre and post each cycle to assess for any change in class I and class II anti-HLA abs (MFIs, titer and cPRA). They will also be collected at two-week intervals during the maintenance phase. Serum samples will be sent to, logged, processed, and stored at the HLA core (Zeevi). Serum samples will be aliquoted in multiple vials to minimize the need of freezing and thawing. Per manufacturer’s instructions, LS1A/LS2A tests will be performed for single-antigen bead (SAB) analysis on the LuminexTM system (One Lambda Thermo Fisher, Canoga Park, CA). The reactions of a given anti-HLA specificity will be expressed as MFI. The drawbacks of using cPRA based on neat MFI values is that i) it only provides a semi-quantitative measure of alloantibody that is impacted by inherent assay limitation and laboratory variability and ii) partial therapeutic effects can be missed because reduction in antibody quantity without completely removing it does not translate to a change in cPRA. One way to overcome these weaknesses is by performing serum dilutions to determine antibody titer and establish the cPRA at various titers. Stepwise dilution of the serum will gradually eliminate antibody positivity and thus decrease the cPRA because the corresponding antigens would no longer be considered unacceptable^7^. We expect that this approach will help stratify patients prior to inclusion in studies and assist in discerning partial therapeutic effects. Titration of serum pre-testing will be performed (1:4, 1:16, 1:64 dilutions) and re-tested for antibody specificity on SAB analysis. We will evaluate the cPRA at different time points and at different dilutions to determine significant changes. A meaningful response following the cycles of treatment will be defined in two ways:

i) cPRA < 50% using an MFI threshold ≥ 4000 based on previous data^15^ or
ii) 50% reduction in MFI and a 2-log reduction in titer as recommended by the Sensitization in Transplantation: Assessment of Risk (STAR) 2022 Working Group^6^.

Antibody evaluation done at the core lab will not be used for clinical decision making at the study sites; those decisions will be made based on antibody evaluations from the HLA laboratories of the individual sites, per their routine practice.

### Statistical and analytical plan

To evaluate the effect of each individual cycle of CFZ+BELA on cPRA, we will fit a linear mixed model with the patient treated as a random effect to control for repeated measures from the same patient. Continuous data (MFI) will be evaluated by mean MFI for class I and class II antibodies respectively with significance determined using student’s T-test. Net MFI reduction will be calculated as: (pre-treatment mean MFI – post treatment mean MFI) / pre-treatment mean MFI. Descriptive statistics in the form of means and standard deviations or medians and inter-quartile ranges will be calculated before and after desensitization cycles for all antibody titers of interest and cPRA. Multivariable analyses of covariance will examine changes in antibody titers and cPRA after controlling for a limited number of clinically important covariates. Paired t-tests or Wilcoxon Signed Rank tests will also be used to examine whether levels of antibody titers and cPRA remain constant between the last desensitization cycle and time of transplant, and to assess reductions in DSA.

### Study funding

This study has been funded by the American Heart Association and Enduring Hearts Translational Research Award in Pediatric Heart Transplantation. All drugs used in the study are covered by patient health insurance.

### Limitations

This study is limited by its modest sample size and the event-driven nature of the PHTS registry, which may under capture negative or missing data such as absent biopsies or unreported rejection. Variability in institutional induction and maintenance immunosuppression practices may introduce heterogeneity not directly addressed by the study design. Long-term post-transplant follow-up and standardized collection of donor-specific antibody data are not currently included but are planned for future phases as the collaborative infrastructure expands.

## Discussion

This multicenter study addresses a critical gap in pediatric heart transplantation by introducing a novel, mechanistically informed desensitization strategy combining CFZ and BELA. Unlike current approaches, which often rely on plasma cell depletion alone, this dual-targeting strategy also inhibits germinal center activity and memory B-cell responses, aiming for more durable antibody suppression. Our protocol incorporates advanced antibody evaluation using serial titrations and cPRA at varying dilutions, offering a more accurate assessment of sensitization risk and transplant eligibility. This is also the first prospective study to utilize the PHTS registry infrastructure, allowing robust outcome tracking across multiple high-volume centers. If effective, this strategy may expand donor options, reduce waitlist mortality, and standardize care for highly sensitized pediatric and young adult HT candidates, providing a platform for future trials and broader clinical adoption.

## Disclosures

This study is funded by the American Heart Association and Enduring Hearts Translational Research Award in Pediatric Heart Transplantation, with Dr. Gokanapudy Hahn as the principal investigator.

## Declaration of competing interest

The authors declare the following financial interests/personal relationships which may be considered as potential competing interests: Lakshmi R. Gokanapudy Hahn reports a relationship with American Heart Association Inc that includes: funding grants. Lakshmi R. Gokanapudy Hahn reports a relationship with Enduring Hearts Foundation that includes: funding grants. If there are other authors, they declare that they have no known competing financial interests or personal relationships that could have appeared to influence the work reported in this paper.
